# Supplementary material for: Plasticity in inhibitory networks improves pattern separation in early olfactory processing
Source: Commun Biol. 2025 Apr 9;8:590. doi: 10.1038/s42003-025-07879-2 (PMC11982548; doi:10.1038/s42003-025-07879-2)
Supplement: Supplementary file 2 — Supplementary Information [file 42003_2025_7879_MOESM2_ESM.pdf]

Plasticity in inhibitory networks improves pattern separation in early olfactory processing - Supplementary Figures

Shruti Joshi[1,2*], Seth Haney[2], Zhenyu Wang[3], Fernando Locatelli[5], Hong Lei[4], Yu Cao[6], Brian Smith[4], Maxim Bazhenov[2*]

[1]Department of Electrical and Computer Engineering, University of California San Diego, USA.
[2]Department of Medicine, University of California San Diego, USA.
[3]Department of Electrical, Computer and Energy Engineering, Arizona State University, USA.
[4]School of Life Science, Arizona State University, USA.
[5]Facultad de Ciencias Exactas y Naturales, Universidad de Buenos Aires, Instituto de Fisiología, Biología Molecular y Neurociencias, CONICET, Buenos Aires, Argentina.
[6]Department of Electrical and Computer Engineering, University of Minnesota, USA.

*Corresponding author(s). E-mail(s): s4joshi@ucsd.edu; mbazhenov@health.ucsd.edu;
Contributing authors: sethdhaney@gmail.com; zwang586@asu.edu; fflocatelli@gmail.com; hong.lei@asu.edu; yucao@umn.edu; brianhsmith@asu.edu;

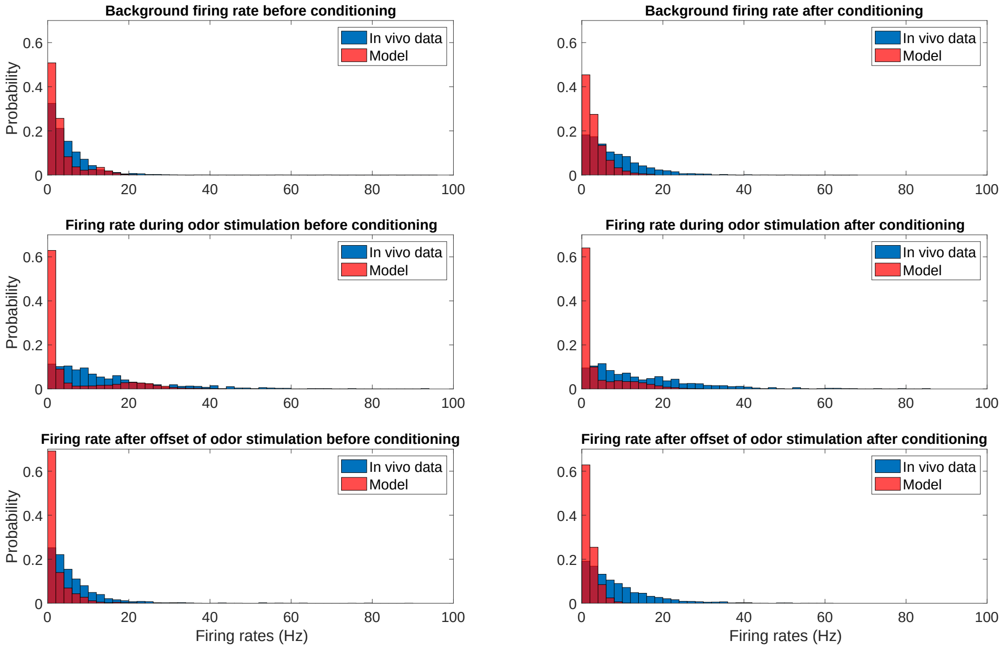

Fig. 1 Histograms of firing rates for in vivo and model PN responses for before and after conditioning. The three rows show firing rates calculated during different time periods. Row 1 shows firing rates for background activity. Row 2 shows firing rates calculated during 500ms of odor stimulation. Row 3 shows firing rates calculated 1s after the offset of the odor stimulation.

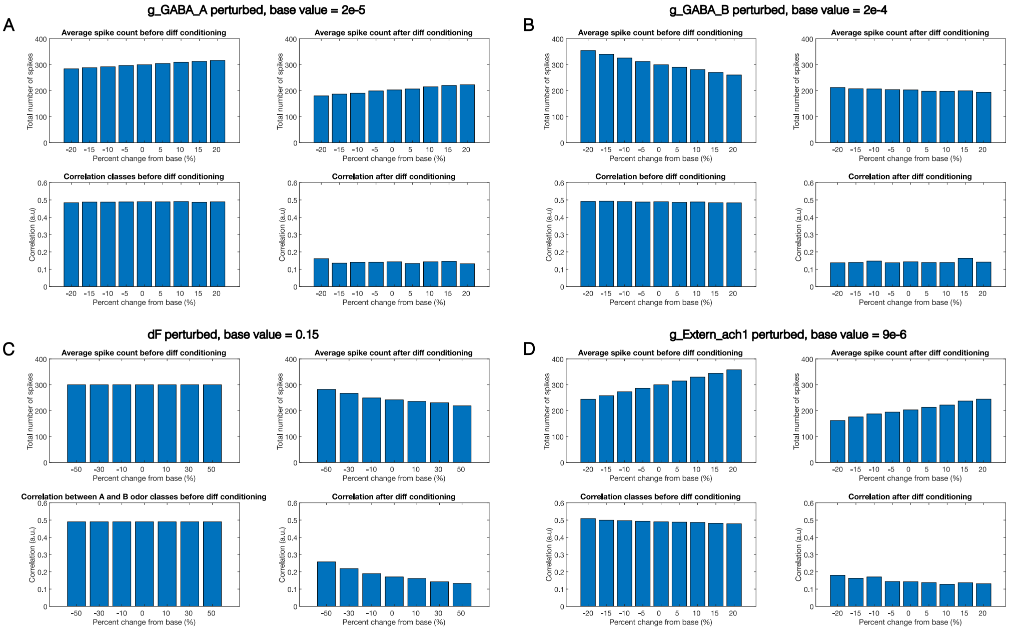

Fig. 2 Parameter sensitivity analysis demonstrates robustness of model behavior. (A-D) Each panel shows the effect of perturbing a different parameter: (A) fast GABA inhibition (g_GABA_A, base value = 2.0e-5), (B) slow GABA inhibition (g_GABA_B, base value = 2.0e-4), (C) facilitation rate (dF, base value = 1.5e-1), and (D) input synaptic conductance (g_Extern_ach1, base value = 9.0e-6). For each parameter, the top row shows the average spike count across all PNs before (left) and after (right) differential conditioning, while the bottom row shows the correlation between odor classes A and B before (left) and after (right) differential conditioning. The x-axis represents the percent change from the baseline parameter value. Despite variations in absolute spike counts, the key finding of reduced correlation between odor representations after differential conditioning remains robust across parameter perturbations, indicating that the model's core behavior does not depend on precise parameter tuning.

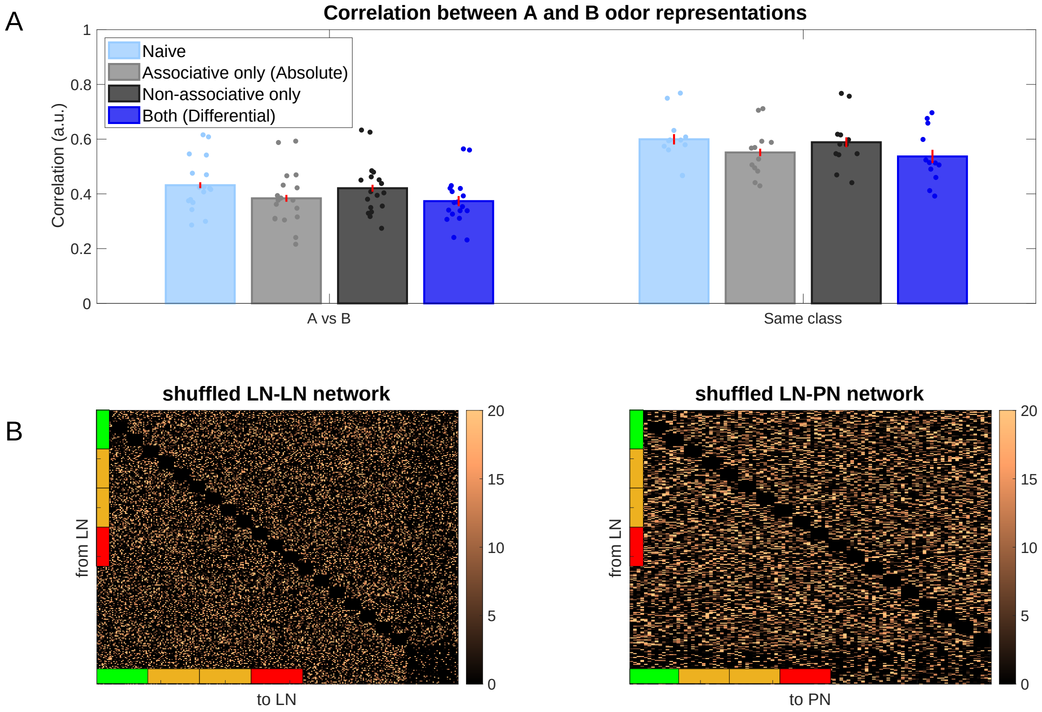

Fig. 3 Analysis of network connectivity structure and odor discrimination. A) Correlation between odor representations comparing naive, associative-only, non-associative-only, and combined (differential) conditioning for odors between different classes (left) and for odors within the same class (right). Error bars show standard deviation. B) Connectivity matrices showing shuffled LN-LN and LN-PN networks after differential conditioning. When the structured connectivity patterns are disrupted through shuffling while maintaining the same total inhibitory strength, the enhanced odor discrimination is lost, demonstrating that the specific spatial organization of inhibitory connections, not just increased inhibition, is essential for improved odor discrimination. Side bars indicate percept identities (green: unique to rewarded odor, orange: shared, red: unique to habituated odor). Color bars show relative synaptic weights.
